# Temporal Bacterial Community Diversity in the *Nicotiana tabacum* Rhizosphere Over Years of Continuous Monocropping

**DOI:** 10.3389/fmicb.2021.641643

**Published:** 2021-05-25

**Authors:** Lang Yan, Wenyou Zhang, Wangjun Duan, Yizheng Zhang, Wen Zheng, Xianjun Lai

**Affiliations:** ^1^Panxi Crops Research and Utilization Key Laboratory of Sichuan Province, College of Agriculture Science, Xichang University, Xichang, China; ^2^China Tobacco Sichuan Industrial Co., Ltd., Chengdu, China; ^3^Sichuan Key Laboratory of Molecular Biology and Biotechnology, College of Life Sciences, Sichuan University, Chengdu, China

**Keywords:** flue-cured tobacco, continuous monocropping, monoculture problems, rhizosphere soil, microbial community

## Abstract

Long-term continuous monocropping negatively influences the physicochemical and biological characteristics of cultivated soil, especially for the economically important crop of flue-cured tobacco that is intolerant to continuous monocropping. The underlying mechanism of soil sickness under continuous monoculture and the temporal dynamic changes over the tobacco life cycle among different monoculture time spans remain poorly characterized. In this study, high-throughput sequencing targeting the 16S rRNA gene phylogenetic marker was performed on 60 soil samples of rhizosphere soil from flue−cured tobacco in the replanting, growth and harvest period across 5, 10, and 20 years of a continuous monocropping system. Bacterial community diversity decreased with the increase in duration of continuous monocropping, and the rhizosphere microbiota was highly dynamic in the harvest period. The random forests algorithm identified 17 taxa as biomarkers and a model was established to correlate root microbiota with continuous monocropping time of flue-cured tobacco. Molecular ecological network analysis elaborated the differences and interactions in bacterial co-occurrence patterns under different monocropping systems. The co-occurrence microbial network was larger in size but there were fewer interactions among microbial communities with the increase in continuous monocropping duration. These results provide insights into the changes of flue−cured tobacco root microbiome diversity in response to continuous monocropping and suggest a model for successional dynamics of the root-associated microbiota over continuous monocropping time and development stage. This study may help elucidate the theoretical basis underlying obstacles to continuous monocropping and could contribute to improving guidance for tobacco production.

## Introduction

Rhizospheres recruit and assemble soil-derived microbial communities, forming a unique microecosystem that is mutually beneficial to plant roots and root-associated microbiota (Edwards et al., 2015, 2018). Metabolites secreted by plant roots provide favorable conditions for growth and reproduction of microorganisms. In return, beneficial microbiota in the rhizosphere convert critical soil nutrients to more usable forms for root assimilation, as well as contributing to pathogen resistance (Edwards et al., 2015; Gao et al., 2019). The rhizosphere is therefore the primary area for signal communication, and material and energy exchanges among plants, soil, and microorganisms (Levy et al., 2018; Rodriguez et al., 2019). Rhizosphere microbial community has been an area of research focus due to the enrichment of copiotrophic microbes in rhizosphere, whereas soils outside the rhizosphere are enriched for oligotrophic microbes (Fierer et al., 2007; Edwards et al., 2018). Recent studies have provided insights into the composition and diversity of rhizosphere and non-rhizosphere soil microbial communities, especially in important model plants and crops such as *Arabidopsis* (Bai et al., 2015), rice (Edwards et al., 2015), and maize (Niu et al., 2017), as well as rhizome crops like sweet potato (Li et al., 2019) and medicinal licorices like Glycyrrhiza (Dang et al., 2020). Furthermore, the increasing abundance of microbiomes has revealed the causative agents of community structure variation in the rhizosphere, for example factors such as genotype and geographical location (Edwards et al., 2015, 2018). However, there is limited information available about how spatiotemporal dynamics of rhizosphere microbiota vary on monthly timescales from seedling to maturity stage of a plant life cycle, and what patterns of change can be expected over years of continuous monocropping for annual plants.

Continuous monoculture negatively influences the physicochemical and biological characteristics of cultivated soil including organic matter, soil enzyme activities, and microbial communities. Along with years of monocropping, constant root secretions in soil may increase the abundance of rhizodepositions, associating with a microflora imbalance between soil and roots that leads to the spread of pathogens and aggravates soil-borne diseases (Yao et al., 2006; Zhou et al., 2011; Liu et al., 2015). Many crops are reported to suffer low yields, high mortality, and degradation of quality due to continuous monocropping obstacles, especially plants of the family *Solanaceae* including tomatoes (Fu et al., 2017), potatoes (Zhao et al., 2020), eggplants (Ikeda et al., 2015), peppers (Xiong et al., 2015), and tobaccos (Wang et al., 2020). Flue-cured tobacco (Virginia tobacco), an economically important crop and the main raw material for the worldwide cigarette industry, is typically intolerant to continuous monocropping (Ren et al., 2015; Wang et al., 2020). The mountainous areas of southwest China have the climatic conditions and geographical preference for regional agro-industrialization that are associated with tobacco leaf quality, and consequently the majority of tobacco in this area is usually cropped in continuous cropping system. Although continuous cropping has seriously affected sustainable development of flue-cured tobacco production (Jian-guo, 2012; Chen et al., 2018), the underlying mechanism of continuous monocropping and soil sickness remains poorly characterized. Based on previous reports, there are four main factors contributing to soil sickness: disorder in physicochemical soil properties, production and accumulation of autotoxins, imbalance of soil microbial communities and deficiencies in soil nutrient content and enzyme activity (Huang et al., 2013; Zhou et al., 2018). Autotoxicity is thought to be a major reason causing continuous cropping obstacle in flue-cured tobacco (Ren et al., 2015). Generally, tobacco more easily generates autotoxic allelopathy than other plants. For example, the extracts of tobacco rhizosphere soil significantly inhibit tobacco seed germination and negatively influence plant growth and development (Jiajun et al., 2017). Soil microorganisms are considered to be key drivers of soil ecosystems, which are critical to many processes including mineral nutrition cyclin, organic matter turnover, soil structure maintenance, and toxin accumulation or removal (Blagodatskaya and Kuzyakov, 2013). Microbial community continuously exposed to root exudates of the same crop under continuous monoculture would potentially enrich the crop−specific microorganisms in soil. Previous studies indicate that long-term continuous cropping leads to changes in soil bacterial community structure and reduced bacterial diversity, indicating that reduced bacterial diversity is another major contributor to the issues associated with continuous cropping (Wang et al., 2017). Due to the decrease in soil microbial community diversity is associated with deficiencies in soil nutrient content and enzyme activity, resulting in yield reductions and poor seedling growth and leaf quality, the diversity of soil microbial community structure could thus be treated as the earliest observable index of soil quality (Ogweno and Yu, 2006; Fuentes et al., 2009; Ren et al., 2015).

Given the probable role that an imbalanced community of root-associated microbiota are responsible for the monocropping obstacles, it is important to understand how root microbiota are assembled and structured over different development stage and whether the temporal shifts are consistent over the years of continuous monocropping. We hypothesized that the long-term practice of continuous cropping would lead to the selection of distinct microbiota communities that were significantly colonized by certain beneficial or pathogenic microorganisms. However, it has yet to be elucidated whether root-associated microbiota vary over the annual life cycle of a crop and whether microbiota assemble in a consistent manner across large timescales (years) of continuous monocropping. Consequently, there were three main objectives in the current study to explore the underlying mechanism(s) of continuous monocropping in flue-cured tobacco: (1) to understand microbial ecological patterns and their relationships with soil physicochemical properties and environmental factors in response to continuous monocropping; (2) to examine the characterized microorganisms as biomarkers in the rhizosphere to predict the prevailing models that reflect the soil conditions during continuous monocropping; and (3) to determine if continuous monocropping affects the root-associated microbiota in different development stages of the crop, and if it does, how the root microbiota change over the full life cycle of the crop among the different monoculture time spans.

To understand temporal dynamic changes in the rhizosphere bacterial community in continuous monoculture, high-throughput 16S rRNA gene sequencing was performed in bulk on rhizosphere soil samples of flue−cured tobacco that was non-cropped (CK) or continuously monocropped for 5, 10, and 20 years (5, 10, and 20 yrCC, respectively) at the same geographical location. In addition, to increase knowledge of the response of bacterial community structure to the continuous cropping of flue−cured tobacco, a spatiotemporal approach was employed to detail the successional progression of the microbiota in the periods of transplanting, growth, and harvest across 5–20 years of a continuous monocropping system. This study provides insights into root microbiome assembly of flue−cured tobacco in response to continuous monocropping and may help elucidate the theoretical basis of the mechanism underlying continuous monocropping obstacles, allowing artificial interventions and replanting improvements of this important crop in the future.

## Materials and Methods

### Tobacco Plant Growth and Sampling

Samples were collected from a commercially long-term cultivated tobacco field, the standardized raw material planting base of flue-cured tobacco named “Kuan&Zhai Garden” established by China Tobacco Sichuan Industrial Co., Ltd. This site is located at Wudongde town, Huidong County, Liangshan, southwest China (longitude/latitude: E102°13′/N26°12′, elevation: 1874 m a.s.l.). There is a long history of flue-cured tobacco cultivation in this area, with some sites having spent more than 30 years under the continuous monocropping system. Soil type in the experimental fields was ACfa (Alumi-Ferric Alisols) according to the FAO-UNESCO Soil Map of the World, and the fields were flat with uniform fertility in the long-term and had compound fertilizer applied at the rate of 750 kg ha^–1^ annually, the formulation of the fertilizer was as follows: N:P_2_O_5_:K_2_O = 10:12:18. Along with the expansion of the cultivated base, fresh fields were constantly being used for flue-cured tobacco cultivation during the past few decades. Samples defined as 5, 10, and 20 yrCC were collected from the rhizosphere of flue-cured tobacco material named “Honghuadajinyuan” (HD) after the fifth, tenth, and twentieth years of continuous monocropping, respectively, and control soil samples defined as CK were collected from non-planting areas around the tobacco-planted areas. Rhizosphere soil of flue−cured tobacco was sampled at three development stages of the crop, including transplanting, growth (70 days after transplanting), and harvest (118 days after transplanting) in each field of the different continuous monocropping systems.

The area of sampling was restricted to a field of 667 m^2^ under each defined year of monocropping and was separated into six regions represented by replications. Each sample was collected from five individual tobacco within a field region of 110 m^2^, at distances of approximately 10 m from each other. To collect rhizosphere soil, roots were firstly shaken to remove large clumps of soil (the clumps were retained), then the roots with tightly adhering soil were placed into 50-mL falcon tubes containing 15 mL autoclaved phosphate-buffered saline (PBS) solution and transported on ice to the laboratory. The roots with soil attached were vortexed in PBS solution with cyclical washing in fresh PBS solution until no soil particles were visible in the solution. All the collected PBS solutions were centrifuged at 12,000 r.p.m. for 10 min, the supernatants were removed, and 10 mL of the resuspended slurry was used for DNA extraction. Large clumps shaken from the roots at each sampling site with six replications were air-dried and analyzed for physical and chemical indexes.

### Analysis of Soil Properties

Air-dried soil samples were passed through a 2-mm sieve and soil pH, organic matter (OM), total nitrogen (TN), nitrate-nitrogen (NO^3–^-N), total phosphorus (TP), available phosphorus (AP), total potassium (TK), and available potassium (AK) were assayed. Soil pH was measured using a pH meter (Mettler Toledo, United States) in a soil water suspension (1:5, w/v) after shaking for 30 min. OM was determined by the potassium dichromate method (Ciavatta et al., 1991). Soil TN and NO^3–^-N were assayed using the procedure reported by Singh et al. (2010). TP and AP were determined according to the method of Ryan et al. (2001), and TK and AK were assayed as described by Zhu et al. (2019).

### DNA Extraction, PCR Amplification, and Sequencing

DNA extractions were performed using the MoBio Powersoil DNA isolation kits following the manufacturer’s instructions. DNA purity was quantified by a NanoDrop spectrophotometer and DNA quality and integrity were assessed by 1% agarose gel electrophoresis. The V3–V4 hypervariable region of the bacterial 16S rRNA gene was amplified using the universal primers 341F and 806R (F5′)-ACTCCTACGGGAGGCAGCAG-3′), (R5′)-GGACTACHVGGGTWTCTAAT-3′) (Caporaso et al., 2011). The resultant PCR products were purified, pooled in equimolar concentrations, and pair-end sequenced using the Illumina MiSeq platform (Illumina, Inc., CA, United States) at Biomarker Technologies Corporation (Beijing, China).

### Sequence Processing and Operational Taxonomic Unit (OTU) Clustering and Filtering

Raw sequence data were demultiplexed using the barcode sequences by in-house Python scripts, then quality filtered and assembled into full contigs using Trimmomatic (Bolger et al., 2014) and FLASH (Magoè and Salzberg, 2011). Raw tags over a 50-bp sliding window at any site with an average quality score lower than 20 were truncated. After filtering tags with a low-quality score (average Phred score of the bases ≤ 20), tags containing ≥3 ambiguous N bases, and tags identified as chimeric sequences in UCHIME (Edgar et al., 2011), the remaining clean tags were clustered into OTUs at a similarity level of 97% using USEARCH (Bokulich et al., 2013). OTUs were aligned to the SILVA database to remove host plastids (Quast et al., 2012). To account for sequencing depth differences between samples, each OTU was divided by the total sequencing depth for each sample and multiplied by 1,000, resulting in a relative abundance in units of per mille. OTUs occurring in less than 5% of the samples were filtered from the OTU table. The Ribosomal Database Project (RDP) classifier tool with a confidence threshold of 0.8 was then used to classify each representative tag into different taxonomic groups (Wang et al., 2007).

### Diversity Analysis and Statistical Analysis

Diversity analysis was conducted using R script. Differential OTU abundance was performed using Wilcoxon rank sum tests based on OTUs with median relative abundance from each group >0.2%, and corresponding *P*-values were corrected for multiple tests using a false discovery rate (FDR) set at 0.05. Alpha rarefaction diversity was calculated a QIIME diversity analyses workflow script core_diversity_analyses.py and unconstrained principal coordinates analysis (PCoA) was performed using the vegan capscale() function in R by specifying an intercept-only model (R Code: capscale[log_2_(RA)-1], and permutational multivariate analysis of variance (PERMANOVA) was conducted using the adonis() function from the vegan package (Oksanen et al., 2013). Redundancy analysis (RDA) was also performed using the vegan package to evaluate the relationships between soil properties and the OTUs of soil bacterial communities. All graphs and plots were generated using the ggplot2 package (Wickham, 2011).

### Generation of Sparse Random Forest Models

To model continuous monocropping years as a function of microbiota composition, a random forest model was generated by regressing the relative abundance of all OTUs against the continuous monocropping years from which the soil samples were collected. Random Forests approach is one of the most robust ensemble machine learning methods for classification and regression. Multiple weak classifiers were assembled to produce a strong classifier. Random Forests tries to grow multiple decision (CART) trees with different samples and different initial variables based on the construction of single classification trees, like a bootstrapping algorithm. Each tree gives a classification. Multiple trees choose the classification with the most votes to perform the final prediction (Ref as follows: https://www.stat.berkeley.edu/∼breiman/RandomForests/cc_home.htm).

Relative abundances of bacterial taxa were classified in the class, order, family, and genus levels against tobacco continuous monocropping years using the randomForest package in R with default parameters (Liaw and Wiener, 2002). The randomForest (importance = TRUE, proximity = TRUE) function was then used to generate the classification model and rank bacterial taxa by their importance in contributing to the accuracy of cropping years prediction by the models.

Ten-fold cross-validation was performed using the rfcv() function in the randomForest R package to remove less featured bacterial taxa and evaluate model performance as a function of inclusion of the top discriminant taxa. The varImpPlot function was used to show the importance of features in the classification. The importance of features and the cross-validation curve were visualized by using the ggplot2 package in R.

### Construction of Bacterial Co-occurrence Network

For network inference, all possible Spearman’s rank correlations between OTUs were calculated, and a valid co-occurrence event was considered to be a robust correlation if the Spearman’s correlation coefficient (r) was both ≥ 0.6 and statistically significant at *P* < 0.01 (Barberán et al., 2012). Nodes in the reconstructed network represent the OTUs at 90% identity, whereas the edges correspond to a strong and significant correlation between nodes. A set of measures including average connectivity (avgK), average clustering coefficient (avgCC), average geodesic distance (avgGD), and modularity were calculated to describe the topology of the resulting network. All statistical analyses were conducted in R using igraph packages. Networks were explored and visualized with the interactive platform Cytoscape v3.8.2 (Shannon et al., 2003).

To define the topological roles of different OTUs (nodes), the connectivity of nodes within (Zi) and among modules (Pi) was calculated using in-house R script according to the functions proposed by Guimera and Amaral (2005) and simplified by Olesen et al. (2007). The node attributes can be divided into four categories according to topological features: (a) Module hubs (generalists, Zi > 2.5 but Pi < 0.62) with high connectivity to nodes within modules; (b) Connectors (generalists, Zi < 2.5 but Pi > 0.62) with many links to the nodes among other modules; (c) Network hubs (supergeneralists, Zi > 2.5 and Pi > 0.62) with great links to the nodes within and among modules; and (d) peripheral nodes (specialists, Zi < 2.5 and Pi < 0.62) with few links and connections to the nodes within modules. Connectors, module hubs, and network hubs are all key microorganisms in the networks.

## Results

### Soil Physicochemical Properties and Field Bacterial Community Structure Variations

The edaphic physicochemical properties among different continuous monocropping systems are shown in Table 1. There was considerable variation in physicochemical properties of the soil samples experiencing 5–20 years of continuous cropping. All soils were alkaline (pH 7.46–8.02), and the CK sample had the highest pH and this was significantly different from the pH of the continuous cropping soil samples. Years of continuous cropping were observed to decrease the content of profitable nutrients, as the amounts of OM, NO^3–^-N, and AK in continuous cropping soil were significantly lower than those in CK samples. However, the AP content was lowest in CK soil and accumulated with the increase in cropping years.

**TABLE 1:** The rhizosphere and bulk soil physical and chemical properties of flue-cured tobacco.

Sample	pH	OM (g/kg)	TN (g/100 g)	NO^3–^-N (mg/kg)	TP (g/100 g)	AP (mg/kg)	TK (g/100 g)	AK (mg/kg)
CK	8.02a	33.8a	0.182a	119a	0.042c	6.5c	1.44c	190a
5 yrCC	7.46c	28.9b	0.073d	108.4b	0.047c	6.7c	1.46c	186b
10 yrCC	7.69b	25.2c	0.150c	83.3c	0.091a	18.2b	1.56b	147c
20 yrCC	7.74b	18.8d	0.166b	60.7d	0.071b	24.4a	1.66a	90d

*OM, organic matter; TN, total nitrogen; NO^3^^–^-N, nitrate-nitrogen; TP, total phosphorus; AP, available phosphorus; TK, total potassium; AK, available potassium. a–d value in the same column with different superscripts are significantly different (P < 0.05) under Shortest Significant Range (SSR) tests.*

A bacterial community profile was generated based on 60 samples collected from the bulk and rhizosphere soil of CK and 5, 10, and 20 years of continuous monocropping, in the periods of transplanting, growth (70 days after transplanting), and harvest (118 days after transplanting) respectively. Through amplification of the V3–V4 region of the 16S rRNA gene followed by Illumina sequencing, 4,973,193 high-quality sequences were obtained from the 60 samples (average, 76,412 clean tags; range, 51,222–158,390 clean tags per sample).

**FIGURE 1:**
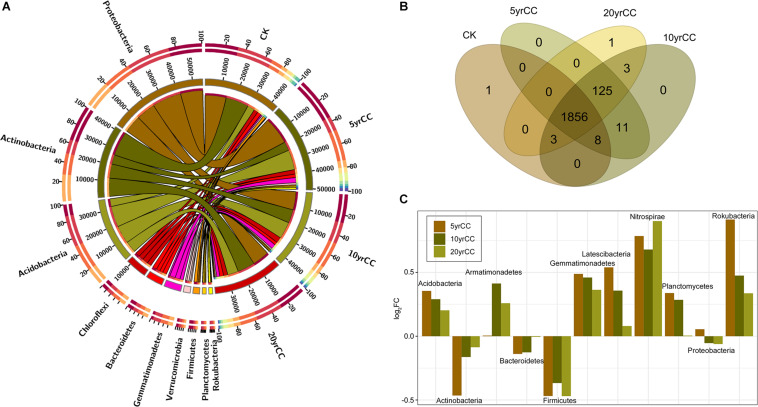
Patterns of relative abundance of bacterial communities among different continuous monoculture time spans. **(A)** Distribution of the 10 most abundant bacterial phyla of soils after different periods of continuous monocropping. The bar length on the outer ring represents the percentage of each phylum in each sample. **(B)** Venn diagram of the number of core- and pan-OTUs among different continuous monoculture time spans. **(C)** Ratio of the log2 fold change in the relative abundance of bacterial taxa in soils from different continuous monocropping time periods and a non-monocropping control. The quantity of OTUs was normalized to unity.

For the bacterial community, there were 2,008 OTUs belonging to 26 phyla, 78 classes, 168 orders, 269 families, and 488 genera in total. At the phylum level, 96.07% of the OTUs were classified in top 10 phyla in terms of relative abundance; these phyla were Proteobacteria, Actinobacteria, Acidobacteria, Chloroflexi, Bacteroidetes, Gemmatimonadetes, Patescibacteria, Verrucomicrobia, Planctomycetes, and Firmicutes. Phylum abundances of the bacterial community across different samples are shown in Figure 1A. Proteobacteria, Actinobacteria, and Acidobacteria accounted for more than 56% of the bacteriome, while the abundance of some common phyla such as Armatimonadetes, Nitrospirae, and Spirochetes were recorded in low abundances in the tobacco rhizosphere. OTUs were further classified into core- and pan-OTUs to identify the common and unique bacterial distribution across samples (Figure 1B). There were 1,856 OTUs (92.43%) shared by all soil samples, while 140 OTUs were newly recruited to the rhizosphere after tobacco planting and were predominantly classified in the phyla Proteobacteria, Bacteroidetes, Cyanobacteria, Patescibacteria, and Gemmatimonadetes. Differences in rhizosphere microbiota under different cropping systems were significant and detectable for the dominant phyla, with Actinobacteria present at higher relative abundance in non-cropping soil (CK) than in continuous cropping samples. The relative abundance of Actinobacteria in 5 yrCC soil samples decreased by 22.03% compared with that in CK samples but gradually increased with the increase in continuous monocropping time. The same pattern of relative abundance was also recorded for the phylum Bacteroidetes. Most phyla had higher relative abundance in continuous cropping soil, for example Acidobacteria and Gemmatimonadetes, although the differences (log_2_FC) between continuous cropping and CK decreased with the increase in continuous monocropping time (Figure 1C).

### Bacterial Diversity Influenced by Continuous Monocropping Time

**FIGURE 2:**
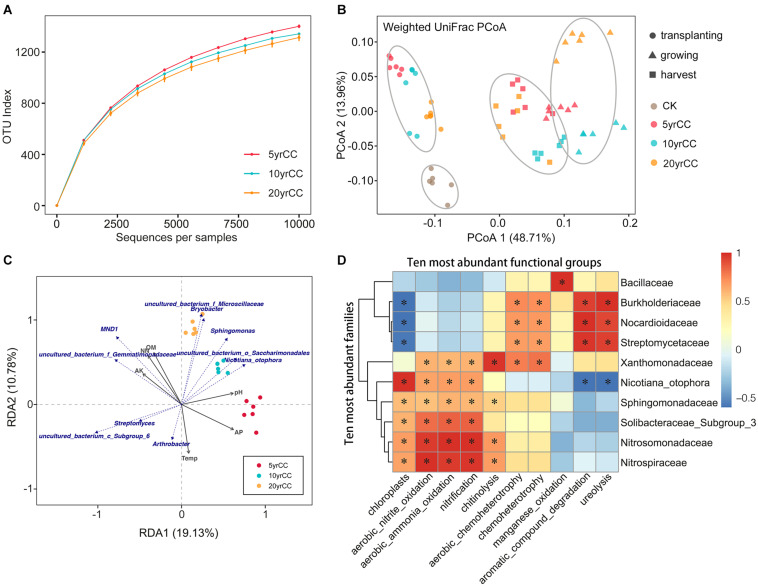
Diversity of bacterial communities at sites after various years of continuous monocropping. **(A)** Rarefaction curves for alpha diversity measures of OTUs comparing microbiota from the continuously cropped soils. Error bars correspond to one standard deviation out from the average (*n* = 6 biological replicates. **(B)** Unconstrained PCoA (for principal coordinates PCo1 and PCo2) with weighted unifrac distance showing that root microbiota in different periods separate in the first axis (*P* < 0.001, PERMANOVA). **(C)** RDA plots of the bacterial communities with respect to environmental variables in the root zone of tobacco. Blue text indicates the bacterial phyla with top 10 abundance, and gray arrows represent different soil properties. **(D)** Heatmap of correlations between bacterial families with ecological functions in rhizosphere soil. Heatmap values ranged from +1 to −0.5. Values above/below zero represent positive/negative correlations between bacterial families and parameters analyzed. *P < 0.05 for the indicated comparisons.

Measurement of within-sample diversity (α-diversity) revealed significant differences among samples exposed to different continuous monocropping times. Rarefaction plots of OTUs, Faith PD and Shannon (*t*-test, *P* < 0.05, Supplementary Table 1) confirm that there was a significant correlation between community diversity and continuous monocropping time. The microbiota from soils with 5 years of continuous monocropping is significantly more diverse than that with 10 and 20 years of continuous monocropping (Figure 2A and Supplementary Figure 1). This indicated that the increase in duration of continuous cropping resulted in recruitment of fewer bacterial species. Moreover, the abundance and diversity of the bacterial communities varied with the different development stages of the flue-cured tobacco; rhizosphere microbiota in harvest period samples had higher diversity than those of the growth period samples (Supplementary Figure 2). These results demonstrated that both the duration of continuous monocropping and the growth and development stage of tobacco itself affected the diversity of rhizosphere bacteria.

To elucidate the degree by which bacterial communities were influenced by the continuous monocropping system and the development stage of tobacco, and which factor(s) could be the underlying driving forces of bacterial community variation in the data, PCoA was conducted based on the weighted UniFrac distance matrix in combination with PERMANOVA. PCoA revealed that the root microbiota of flue−cured tobacco formed three distinct clusters with the development stages, which separated along the first coordinate (Figure 2B). This indicated that the largest source of variation in the root microbiota was proximity to the growing periods of tobacco. PERMANOVA of pairwise distances between bacterial communities indicated that the microbiota differed significantly (*R*^2^ = 0.58, *P* < 0.001). Divergences in β-diversity were still identified among 5, 10, and 20 yrCC rhizosphere samples within each cluster separated by distinct growing period, and this observation was supported by the PERMANOVA statistic (*R*^2^ = 0.208, *P* < 0.001). This suggested the composition of the tobacco rhizosphere bacterial community was affected by the development stage time-spans in the full life-cycle of the plants and marginally influenced by the continuous monocropping time-spans.

Relationships between soil properties and bacterial community composition significantly differed among the investigated cropping systems. Climate and soil physicochemical properties (soil temperature, pH, OM, TN, NO^3–^-N, AP, and AK) were explanatory factors determining the observed clustering pattern of bacterial communities for different continuous monocropping times and development stages. RDA was conducted to determine the main environmental factors responsible for the variation of bacterial phylogenetic structure (Figure 2C). Only the bacterial community in the rhizosphere represented by the growing period are shown. Soil pH and AP were identified as primary explanatory factors responsible for the observed clustering pattern in the 5 yrCC rhizosphere bacterial community. In addition, bacteria of the order Saccharimonadales had a significant influence on the 10 yrCC rhizosphere bacterial community structure, and bacteria in Microscillaceae and Bryobacter played an important role in shaping the community structure of the 20 yrCC rhizosphere.

In order to clarify the changes in bacterial community functionality with continuous cropping duration, we further predict the ecological functions of the rhizosphere soil bacterial community during continuous cropping of flue-cured tobacco. Forty-seven ecological functions were annotated by FaProTax software, in which chemoheterotrophy (33.87% ± 1.02%), aerobic chemoheterotrophy (31.63% ± 1.37%), chloroplasts (5.93% ± 2.32%), nitrification (4.15% ± 2.14%), aerobic ammonia oxidation (2.77% ± 1.17%), were the predominant functional groups in the rhizosphere soil of flue-cured tobacco (Supplementary Figure 3). Furthermore, significantly positive relationships were identified between families and functions. The functionalities of ten most abundant families were substantially divided into two groups, one of which mainly associated with chemoheterotrophy and other families mainly participate into nitrogen metabolism. As shown in Figure 2D, Burkholderiaceae, Nocardioidaceae, and Streptomycetaceae were positively correlated with aerobic chemoheterotrophy, chemoheterotrophy, aromatic compound degradation and ureolysis. These families were negatively related to the functional groups which were positively correlated with other bacterial families. For example, Sphingomonadaceae, Solibacteraceae, Nitrosomonadaceae, and Nitrospiraceae were positively correlated with aerobic nitrite oxidation, aerobic ammonia oxidation, nitrification (all *P* < 0.05).

### Predictive Model by Subsets of the Microbiota Biomarkers

**FIGURE 3:**
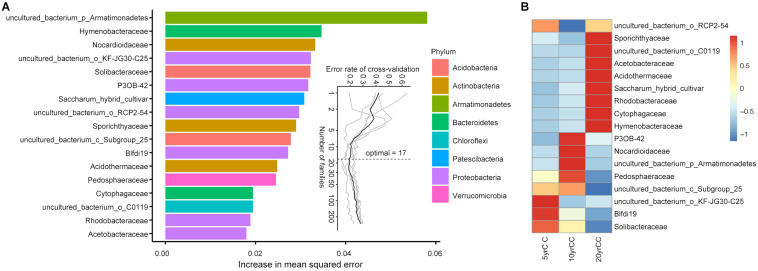
Random-forest model detects bacterial taxa representing the biomarkers across corresponding continuous monocropping time periods of flue-cured tobacco. **(A)** The top 17 bacterial families were identified by applying random-forest classification of the relative abundance of the root microbiota in different monocropping years. Biomarker taxa are ranked in descending order of importance to the accuracy of the model. The inset represents 10-fold cross-validation error as a function of the number of input families used to differentiate biomarker taxa in order of variable importance. **(B)** Heatmap showing the relative abundances of the top 13 predictive biomarker bacterial families against continuous monocropping time periods.

To test whether root microbiota members can be used as biomarkers to differentiate the soil under different years of a continuous monocropping system, the relative abundance of root bacteria at the class, order, family, and genus levels were further regressed against continuous monocropping years of tobacco in the field using the Random forest machine learning algorithm. Using samples collected in the transplanting period, the model established in this study could correlate root microbiota composition with continuous monocropping years. In relation to different cropping times, the model based on bacterial family level explained 74.61% variance of root microbiota classification, the highest within the four tested taxonomic levels. Based on the model, a 10-fold cross-validation with five repeats was performed to evaluate the importance of indicator bacterial families. The minimum cross-validation error curve was obtained when the 17 most relevant families were chosen, which were defined as the biomarker taxa. The top 17 bacterial taxa at the family level across corresponding continuous monocropping time of flue-cured tobacco, in order of time-discriminatory importance, is shown in Figure 3A. “Uncultured bacteria in Armatimonadetes” was increased the maximum squared error in the model, indicating this bacterial taxon is most important for the accuracy of the predicted model.

Most biomarker taxa showed high relative abundance in the corresponding continuous monocropping time of flue-cured tobacco in the field. Solibacteraceae, Myxobacteria (Blfdi19), and KF-JG30-C25 (uncultured bacterial member in Gammaproteobacteria) were defined as the featured biomarkers in soil continuously monocropped for 5 years (Figure 3B). Pedosphaeraceae, Nocardiaceae, Myxobacteria (P3OB-42), and uncultured bacteria in Armatimonadetes started to accumulate in a high relative abundance after 10 years of continuous monocropping. Compared with the two systems above, featured biomarkers in the soil after 20 years of continuous monocropping included Hymenobacteraceae, Cytophagaceae, Rhodobacteraceae, Acidothermaceae, Acetobacteraceae, and Sporichthyaceae, which remained at high levels during long-term continuous monocropping.

### Patterns of Interactions in Bacterial Co-occurrence Network Under Different Cropping Systems

**FIGURE 4:**
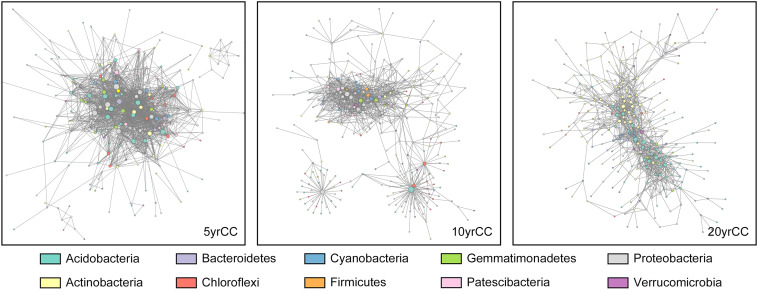
Overview of continuous cropping network in rhizosphere soil samples after continuous monocropping for 5 years (5 yrCC), 10 years (10 yrCC), and 20 years (20 yrCC). The nodes represent OTUs and edges stands for a strong (Spearman’s ρ > 0.6) and significant (*P* < 0.01) correlation. There were 899 positive and 820 negative interaction among 1,719 nodes in 5 yrCC while 1,093 and 1,088 positive interactions and only 2 and 74 negative interactions in 10 and 20 yrCC, respectively. Nodes in different colors belong to the top 10 bacterial phyla in the networks.

Differences and interactions in bacterial co-occurrence patterns under different cropping systems were further revealed by co-occurrence network analysis at the phylum level (Figure 4). Topological properties of the networks showed significant differences in bacterial co-occurrence patterns among 5, 10, and 20 yrCC (Table 2). The 5 yrCC bacterial co-occurrence network had the lowest number of nodes at 249 but the highest number of edges at 1,719 and the highest average degree of 13.807, implying complicated interactions and regulations existed among the hub nodes. The hub nodes with max degree, OTU484 belonging to Nitrosomonadaceae, Gammaproteobacteria, regulated the occurrence of 81 bacteria in the network. As expected, bacterial co-occurrence patterns became relatively simple during long-term continuous monocropping. The number of nodes increased to 386 and 413 in the 10 and 20 yrCC bacterial co-occurrence networks, respectively, with relatively lower edges of 1,095 and 1,162, respectively. In comparison with the intricate co-occurrence patterns in the 5 yrCC system, the average degree in 10 and 20 yrCC networks decreased to 5.67 and 5.627, respectively, and the hub nodes with max degree in the 20 yrCC network, represented by OTU154 (member of Rhodocyclaceae, Gammaproteobacteria) and OTU82 (member of Acidobacteria), only regulated 35 related bacterial occurrences.

**TABLE 2:** Topological properties of bacterial communities in different cropping systems.

Sample (yrCC)	Nodes	Edges	avgK	avgCC	avgGD	M (No of module)	Nodes with max degree
5	249	1,719	13.807	0.312	2.818	0.305 (17)	OTU484 (81)
10	386	1,095	5.67	0.171	5.031	0.514 (32)	OTU34 (55)
20	413	1,162	5.627	0.229	5.405	0.571 (44)	OTU154/OTU82 (35)

*avgK, average connectivity; avgCC, average clustering coefficient; avgGD, average geodesic distance; M, modularity.*

As shown in the Figure 4, network in the 5 years monocropping system recruited various kinds of bacterial taxa, at least of which bacterial in Acidobacteria, Actinobacteria, Proteobacteria, Cyanobacterial, Gemmatimonadetes and Chloroflexi participated in the regulation as the hub nodes. However, the bacterial co-occurrence network appeared to separate into three distinct sub-networks, and bacteria in Proteobacteria, Acidobacteria, and Chloroflexi comprised the main hub nodes for the sub-networks. The composition of the 20 yrCC co-occurrence network showed a uniform pattern in bacterial diversity, in which only members of Actinobacteria and Acidobacteria were identified as the hub nodes. This reduced complexity in the bacterial co-occurrence network with the increase in duration of continuous cropping demonstrated that relatively unitary taxa and a stabilizing co-occurrence pattern played pivotal roles in the long-term continuous cropping system.

**FIGURE 5:**
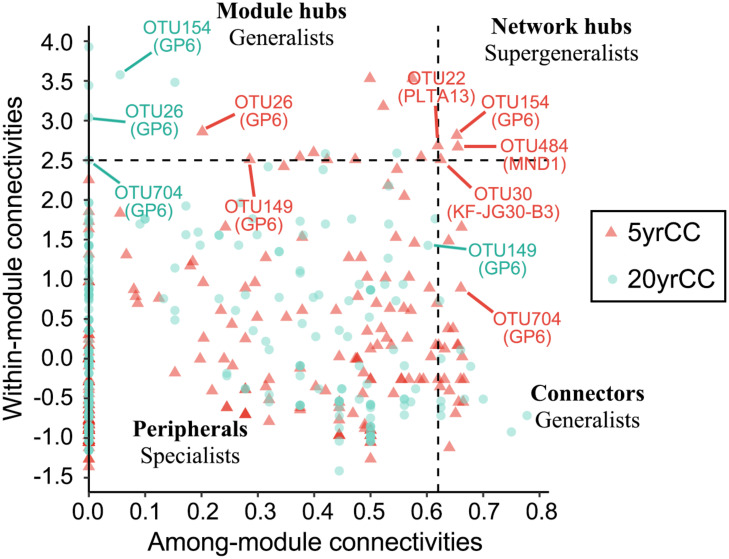
Zi-Pi plot showing the distribution of OTUs based on their topological roles. Each symbol represents an OTU in short-term (5 years) and long-term (20 years) continuous monocropping networks. The threshold values of Zi and Pi for categorizing OTUs were 2.5 and 0.62, respectively, as previously reported (Olesen et al., 2007). All OTUs identified as generalists and co-existing in both groups are labeled.

To identify keystone microorganisms in the networks, the connectivity of OTUs (nodes) within (Zi) and among (Pi) modules were calculated and the OTUs were classified into peripherals, connectors, module hubs, and network hubs according to the threshold values of 2.5 (Zi) and 0.62 (Pi) proposed by Guimera and Amaral (2005) and simplified by Olesen et al. (2007; Figure 5 and Supplementary Figures 4, 5). As shown in the Figure 5, the majority of OTUs were peripherals with few links and connections to the nodes within and among modules; 45% of OTUs for the 5 yrCC network and 69% for the 20 yrCC network had no links to other nodes outside modules (Pi = 0). Compared with the long-term continuous monocropping system of 20 years, the 5 yrCC recruited more generalists, which bridge different nodes within their own modules and/or among different modules, categorized as module hubs of 10 and connectors of 24. Within the 5 yrCC network, four nodes were classified as network hubs (supergeneralists, including OTU154, OTU484, OTU22, and OTU30), whereas no node acted as supergeneralist in the 20 yrCC network, indicating the complicated and extensive regulation present in the short-term continuous monocropping system.

## Discussion

Continuous monocropping causing dynamic disturbance and transformation of soil microorganisms has been reported in previous studies on various crops (Dang et al., 2020; Lei et al., 2020). The current study observed that rhizosphere bacterial diversity of flue-cured tobacco was highly correlated with the time span of continuous monoculture. In general, the diversity decreased with the increase in duration of continuous cropping (Figure 2A). This finding was in agreement with previous reports that cropping systems have significant effects on soil bacterial abundance (Li et al., 2019). However, it was proven not to be the case when the development stage of the plants was taken into consideration. In bulk soil in the replanting period, the richness of bacterial communities increased with the increase in duration of continuous cropping, whereas the values decreased significantly with the increase in years of monocropping when analyzing the rhizosphere soil samples at the harvest stage (Supplementary Figure 6). One explanation for this could be that retaining and harvesting the same crop for a long period of time led to the formation of a relatively stable soil environment and the accumulation of soil nutrients that promoted soil bacterial richness (Wang et al., 2020). However, the available resources of microbiomes for roots as well as the plant-microbial interactions mediated by the soil physicochemical conditions and root exudates were increasingly scarce over years of continuous monocropping, resulting in the significant decrease in diversity indices in the rhizosphere soil of growth and harvest periods (Arafat et al., 2017; Li et al., 2019). The development stage of the plants affected the communities of plant microbiota, and this was also proven by the RF models showing that biomarkers predicted in different group samples of growth period had distinct colonizing microbes (Figure 3 and Supplementary Figures 7, 8). Previous studies reported that root microbiota composition varies with both chronological age and the developmental stage of rice (Edwards et al., 2018), and factors in root exudates might account for some of the shifts observed over the life cycle of *Arabidopsis* and tomato (Davey and Van Staden, 1976; Chaparro et al., 2013). Therefore, attention should be paid to the phase of plant growth of the selected crop in future studies of continuous monocropping obstacles.

In the present study, network analysis was employed to analyze the microbial interactions and co−occurrence patterns between soil bacteria in different tobacco cropping systems. Structures and compositions of the microbial networks varied under prolonged tobacco monoculture; the 20 yrCC microbial network was larger but had fewer interactions among microbial communities compared with the 5 yrCC network. This suggested that microbe-microbe interactions weakened with the increased duration of continuous monocropping, in terms of exchanges of messages, energy, and nutrients among different communities (Lu et al., 2013; Chen et al., 2018). Conversely, this pattern might mean the microorganisms cooperate better to deal with environmental impacts in the soils without continuous monocropping obstacles, since compared with the 20 yrCC network, the 5 yrCC network had far more complicated interactions, recruited more types of bacterial phyla, and most OTUs aggregated into big modules with closer connectivity and organization to each other. Regardless of specific functions, the presence of more highly connected OTUs is beneficial for the rhizosphere community (Chen et al., 2018). Analogous to a human society, the presence of more interrelated individuals and a more clustered topology structure promote highly efficient transactions and cooperation in society.

Based on the results of the current study, a conceptual conclusion was formed as to the major cause(s) leading to the shift of community structure in soils under prolonged tobacco monocropping. The Zi-Pi relationship of each individual OTU revealed that four supergeneralists existed in the network of short-term continuous monocropping (5 yrCC network) as well as 34 generalists (10 module hubs and 24 connectors) connected within and among modules respectively. However, in the long-term continuous monocropping system (20 yrCC network), the number of generalists decreased to 21 and consisted of 8 module hubs and 13 connectors. This suggested that the roles of some nodes shifted in the two networks and that prolonged monocropping changed the ecological roles of key microorganisms in soils, especially decreasing the generalists across different modules. As previously reported, more generalists in networks are favorable for maintaining the balance of microbial communities and are important for promoting exchanges of energy, information, and materials among species in networks (Olesen et al., 2007). Compared with specialists that have specific nutritional requirements and only survive within restricted habitats, generalists can utilize a wide range of nutritional sources and are thus distributed broadly across the soil ecosystem habitats (Olesen et al., 2007; Deng et al., 2012; Tao et al., 2018). Therefore, the decrease in quantity of generalists and supergeneralists after long-term continuous cropping could be viewed as a predominant factor leading to the continuous−cropping obstacle of flue-cured tobacco.

Five nodes classified as generalists were present in both 5 yrCC and 20 yrCC networks and four of these nodes belonged to subgroup 6 of Acidobacteria (Figure 5), demonstrating the important functions of these type of bacteria in microbial community regulation. Some subgroups (Gp4 and Gp6) of Acidobacteria are particularly abundant in soils with a high SOC (soil organic carbon) level (Li et al., 2017), and their ability to decompose organic matter has been reported previously (Rawat et al., 2012; Tveit et al., 2014). The comparison of generalists in both networks focused on the nodes identified as generalists in the 5 yrCC network but which were specialists in the 20 yrCC network, possibly due to poor nutrients and excessive toxic elements occurring with the increase in continuous cropping years. For example, OTU484 was the node with max degree and identified as a supergeneralist in the 5 yrCC network, and was identified a specialist in the 20 yrCC network. This OTU belonged to the family Nitrosomonadaceae, a monophyletic group within the Betaproteobacteria that is important in the nitrification (oxidization of ammonium ions into nitrite) process of the nitrogen cycle. Ammonia oxidation is also carried out by organisms of the Gammaproteobacteria, some nodes of which were identified as supergeneralist (OTU22) and generalist (OTU327, OTU368, OTU267, OTU46, and OTU1168) in the 5 yrCC network but also as a specialist in the 20 yrCC network. Conversely, one third of the generalists belonged to the Actinobacteria in the 20 yrCC network, while there was only one generalist of Actinobacteria in the 5 yrCC network. This demonstrated that the introduction and regulation of Actinobacteria could have been a key and beneficial factor for maintaining stability of microbial networks under long-term continuous cropping. Furthermore, prolonged monocropping having significantly negative influences on the soil microbial community is supported by a previous report that Actinobacteria were more enriched in suppressive soil than in conducive soil and were the most dynamic taxa in suppressive soil amended with the soil-borne pathogen (Mendes et al., 2011). More detailed studies of these types of keystone functional taxa are needed in the future to help contribute to improve the soil environment after long-term continuous cropping of flue-cured tobacco.

## Data Availability Statement

The datasets presented in this study can be found in online repositories. The names of the repository/repositories and accession number(s) can be found below: https://www.ncbi.nlm.nih.gov/, PRJNA684032.

## Author Contributions

LY, WYZ, and XL designed the research. WYZ and WD prepared the plant samples. LY extracted the DNA and amplified the V3–V4 region of the 16S rRNA gene. LY, WZ, and XL performed the data analysis. LY and WYZ wrote the initial draft. YZ, WD, and XL revised the manuscript. All authors read and approved the final manuscript.

## Conflict of Interest

WD is employed by the company China Tobacco Sichuan Industrial Co., Ltd. The remaining authors declare that the research was conducted in the absence of any commercial or financial relationships that could be construed as a potential conflict of interest.

## Supplementary Material

Supplementary Figure 1 Rarefaction curves for alpha diversity measures of Shannon and Faith PD comparing microbiota from the continuously cropped soils. Error bars correspond to one standard deviation out from the average (*n* = 6 biological replicates).

Supplementary Figure 2 Rarefaction curves for alpha diversity measures of OTUs and Faith PD comparing microbiota from three growth periods of flue-cured tobacco. Error bars correspond to one standard deviation out from the average (*n* = 6 biological replicates).

Supplementary Figure 3 Distribution of bacterial ecological function in the FaProTax database for different continuous cropping durations. Values represent means of biological replicates (*n* = 4). The 10 most abundant bacterial functional groups are shown.

Supplementary Figure 4 Zi-Pi plot showing the distribution of OTUs based on their topological roles. Each symbol represents an OTU in the 5- and 10-year continuous monocropping networks.

Supplementary Figure 5 Zi-Pi plot showing the distribution of OTUs based on their topological roles. Each symbol represents an OTU in the 10- and 20-year continuous monocropping networks.

Supplementary Figure 6 Rarefaction curves for alpha diversity measures of OTUs, Faith PD, and Shannon comparing microbiota from the continuously cropped soils in replanting (upper panel), growth (middle panel), and harvest period (lower panel), respectively. Error bars correspond to one standard deviation out from the average (*n* = 6 biological replicates).

Supplementary Figure 7 Random-forest model to detect bacterial taxa representing the biomarkers across corresponding continuous monocropping time spans of flue-cured tobacco as detected by the rhizosphere bacterial community in the period of tobacco growth.

Supplementary Figure 8 Random-forest model to detect bacterial taxa representing the biomarkers across corresponding continuous monocropping time spans of flue-cured tobacco as detected by the rhizosphere bacterial community in the period of tobacco harvest.
