# Supplementary figures and images for: Temporal Bacterial Community Diversity in the Nicotiana tabacum Rhizosphere Over Years of Continuous Monocropping
Source: Front Microbiol. 2021 May 25;12:641643. doi: 10.3389/fmicb.2021.641643 (PMC8186668; doi:10.3389/fmicb.2021.641643)

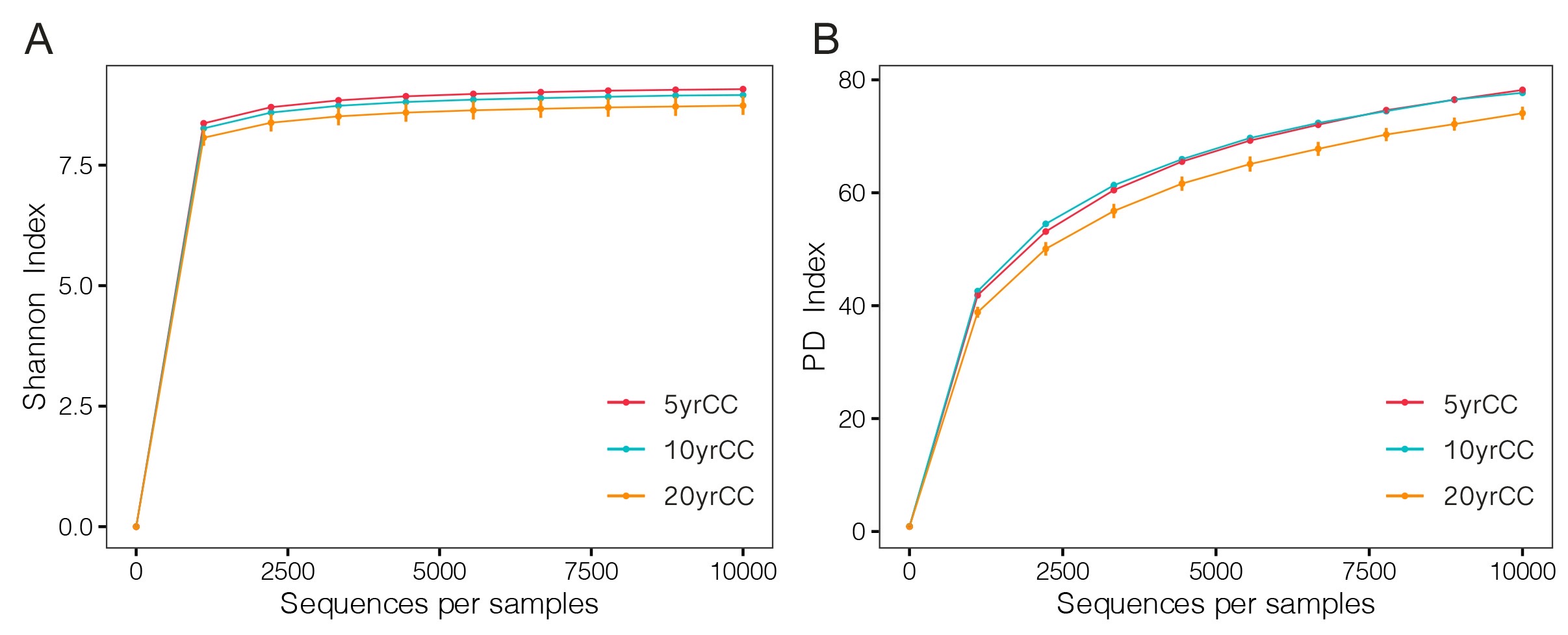

Supplement: Supplementary Figure 1 — Rarefaction curves for alpha diversity measures of Shannon and Faith PD comparing microbiota from the continuously cropped soils. Error bars correspond to one standard deviation out from the average (n = 6 biological replicates). [file Image_1.JPEG]

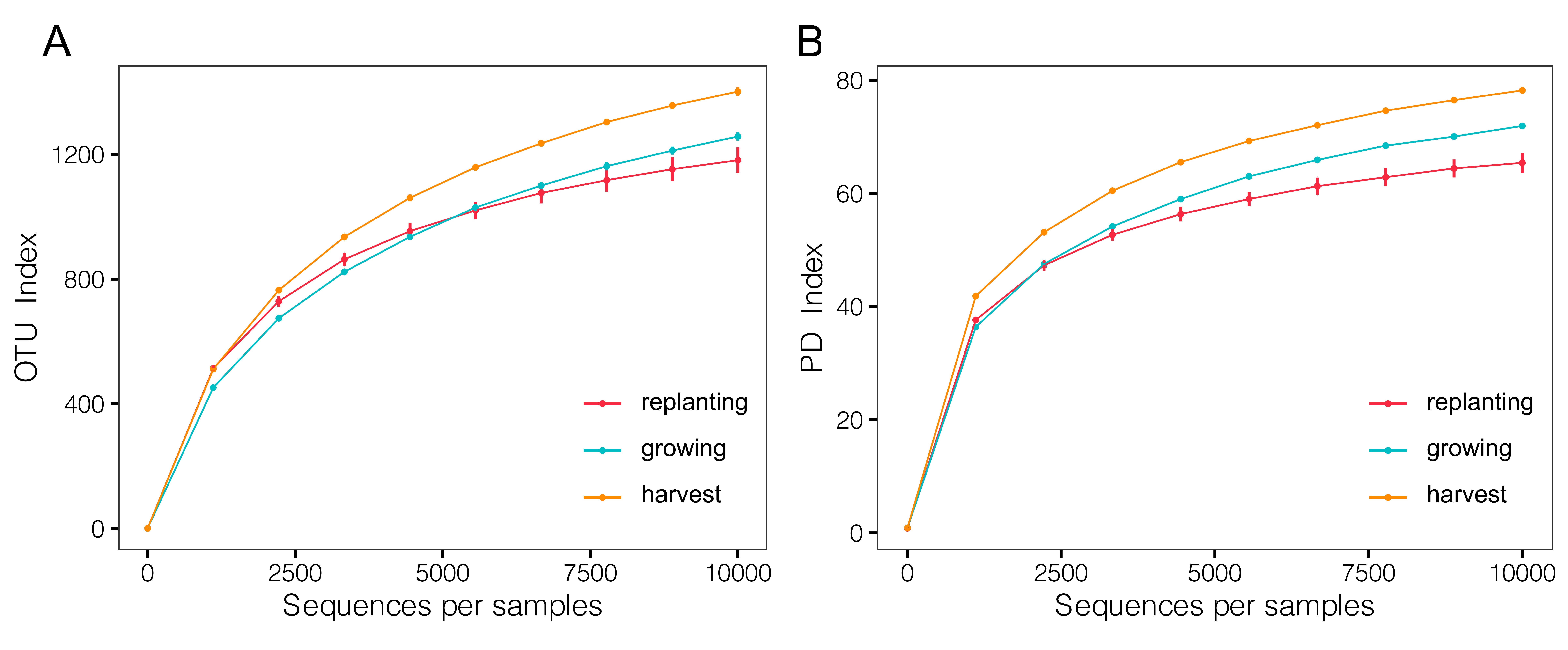

Supplement: Supplementary Figure 2 — Rarefaction curves for alpha diversity measures of OTUs and Faith PD comparing microbiota from three growth periods of flue-cured tobacco. Error bars correspond to one standard deviation out from the average (n = 6 biological replicates). [file Image_2.JPEG]

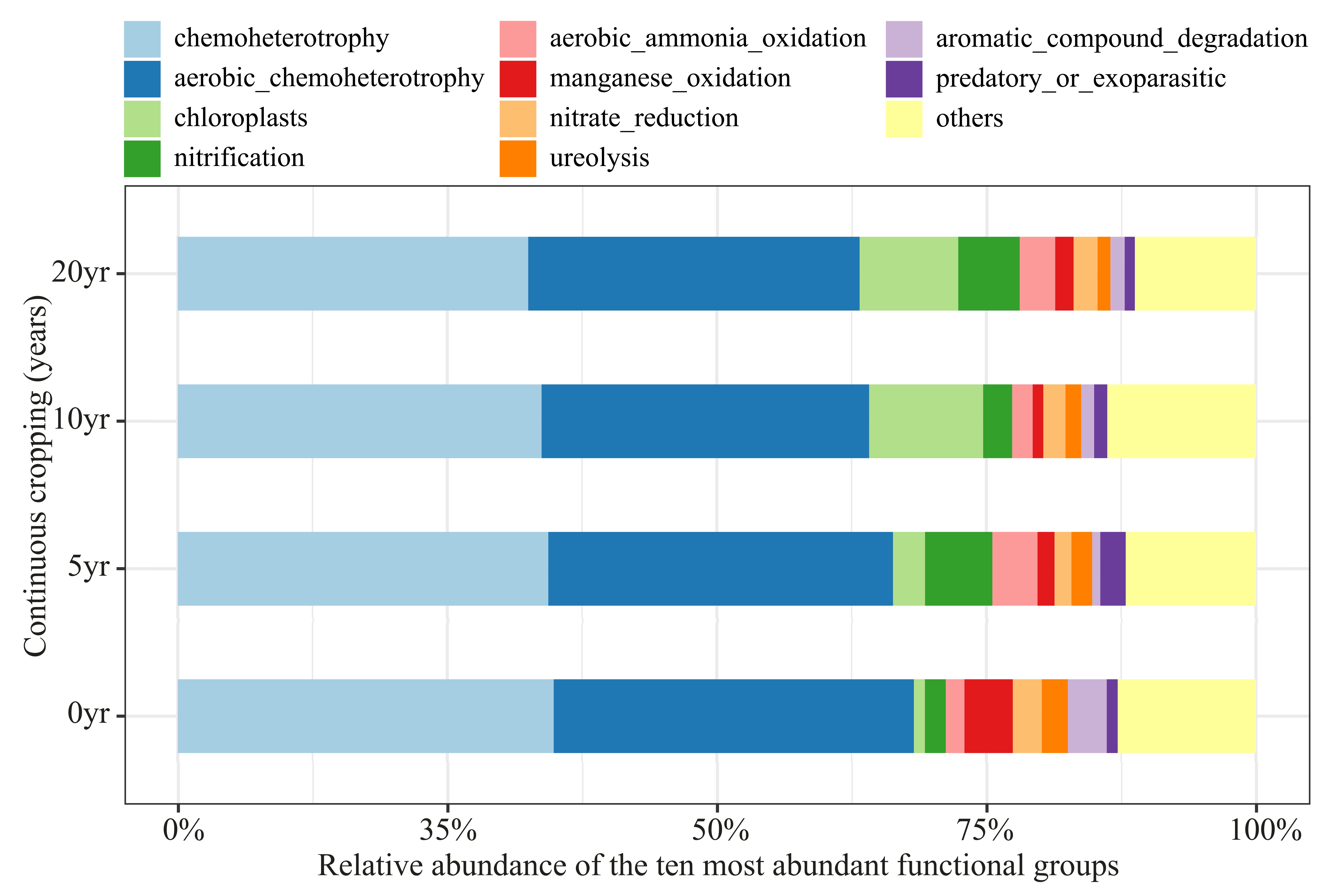

Supplement: Supplementary Figure 3 — Distribution of bacterial ecological function in the FaProTax database for different continuous cropping durations. Values represent means of biological replicates (n = 4). The 10 most abundant bacterial functional groups are shown. [file Image_3.JPEG]

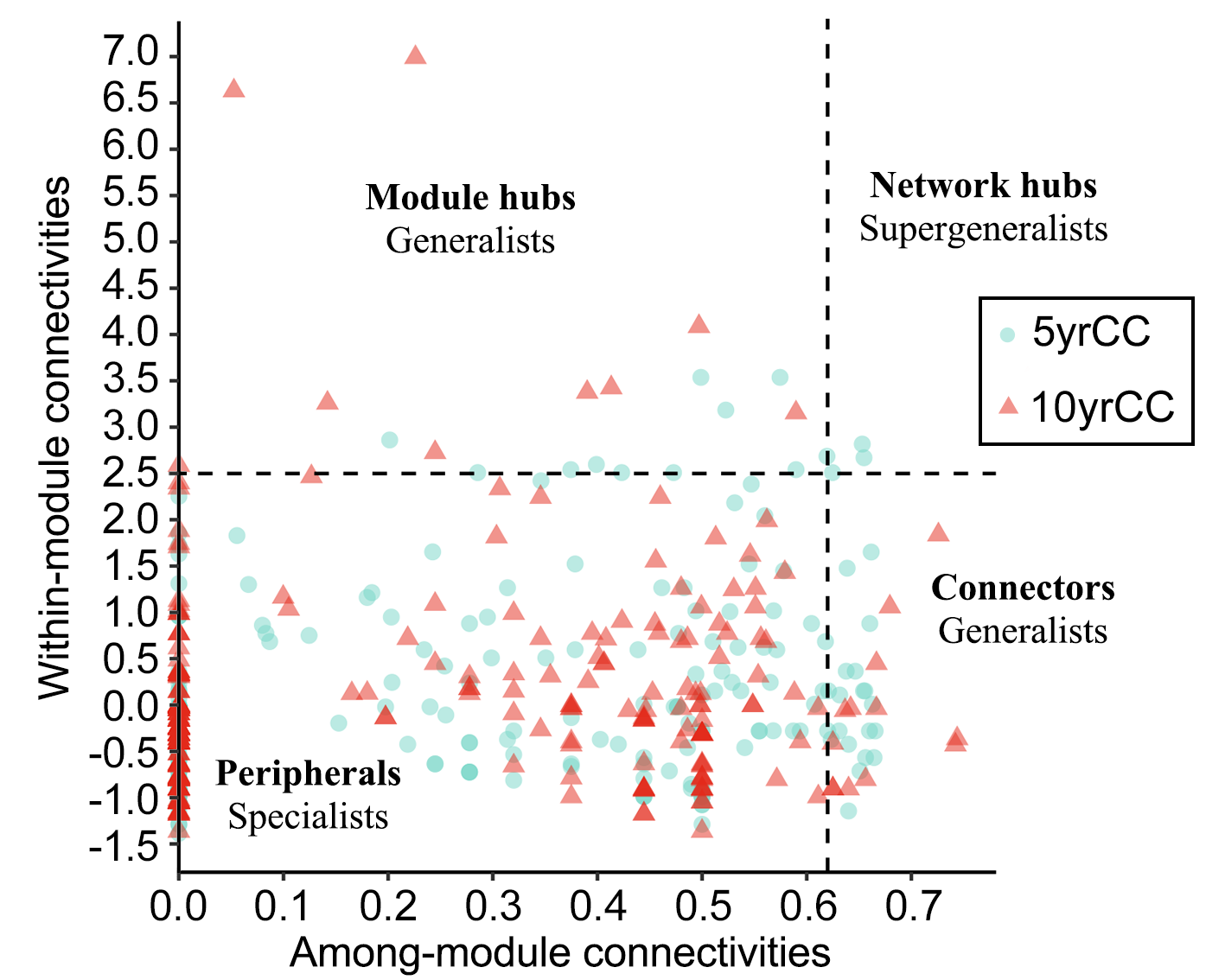

Supplement: Supplementary Figure 4 — Zi-Pi plot showing the distribution of OTUs based on their topological roles. Each symbol represents an OTU in the 5- and 10-year continuous monocropping networks. [file Image_4.TIF]

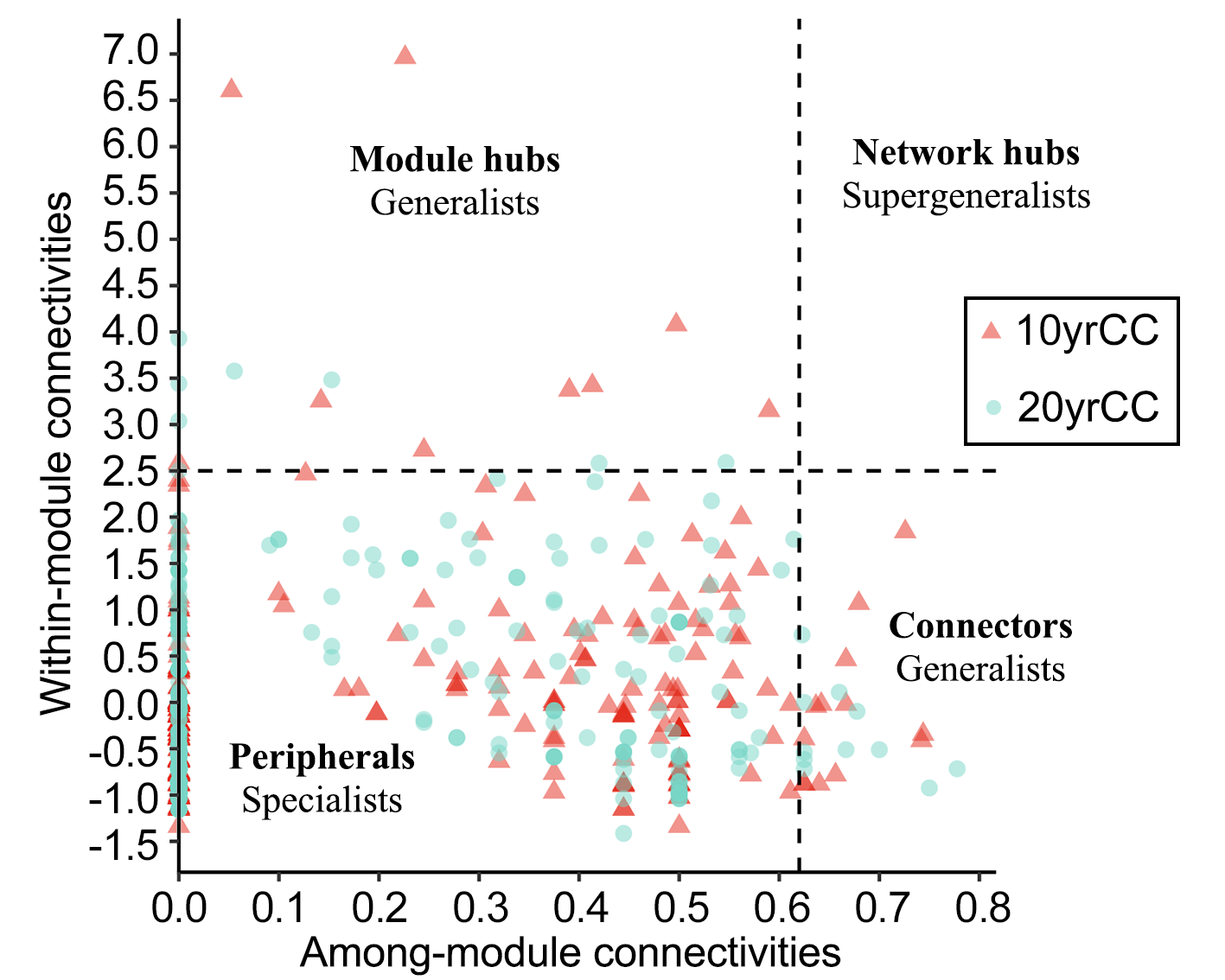

Supplement: Supplementary Figure 5 — Zi-Pi plot showing the distribution of OTUs based on their topological roles. Each symbol represents an OTU in the 10- and 20-year continuous monocropping networks. [file Image_5.TIF]

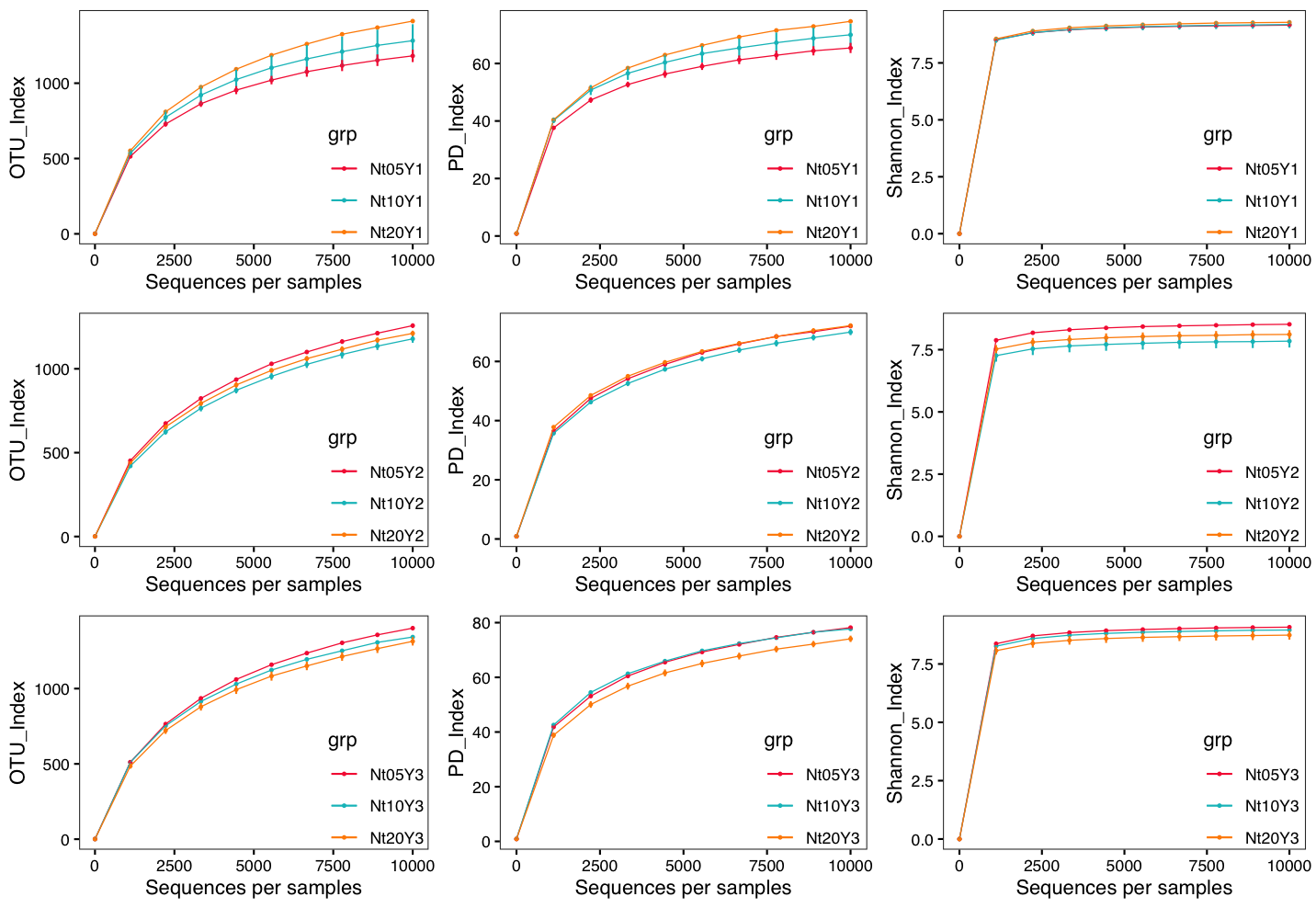

Supplement: Supplementary Figure 6 — Rarefaction curves for alpha diversity measures of OTUs, Faith PD, and Shannon comparing microbiota from the continuously cropped soils in replanting (upper panel), growth (middle panel), and harvest period (lower panel), respectively. Error bars correspond to one standard deviation out from the average (n = 6 biological replicates). [file Image_6.PNG]

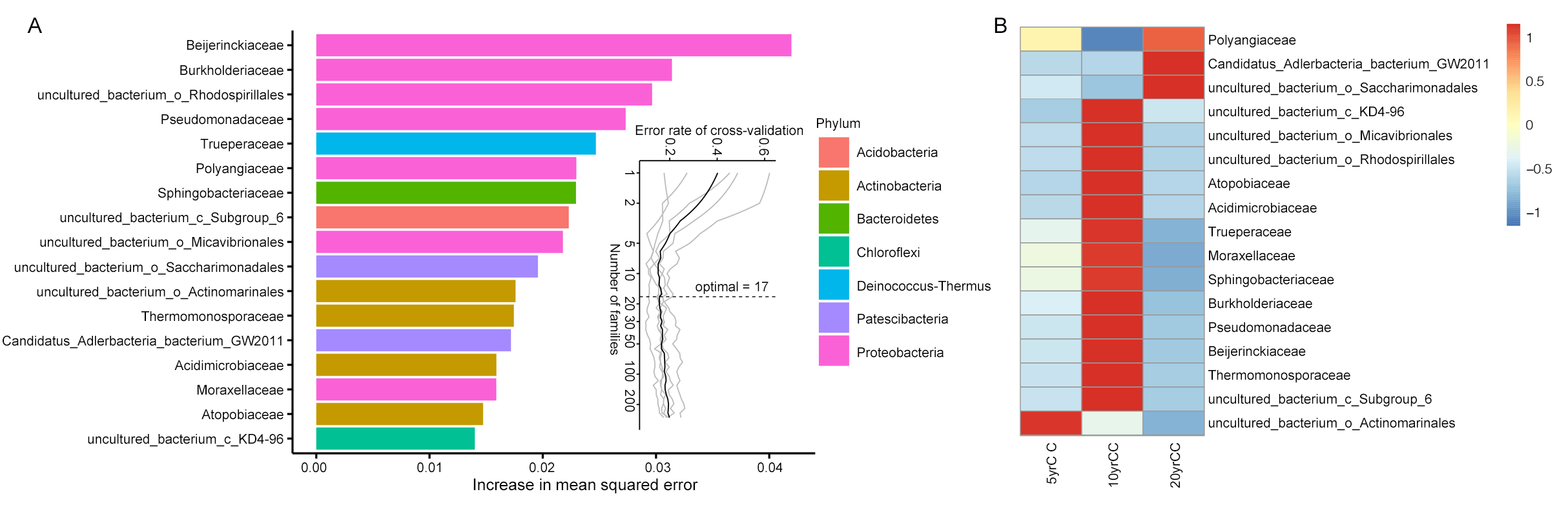

Supplement: Supplementary Figure 7 — Random-forest model to detect bacterial taxa representing the biomarkers across corresponding continuous monocropping time spans of flue-cured tobacco as detected by the rhizosphere bacterial community in the period of tobacco growth. [file Image_7.TIF]

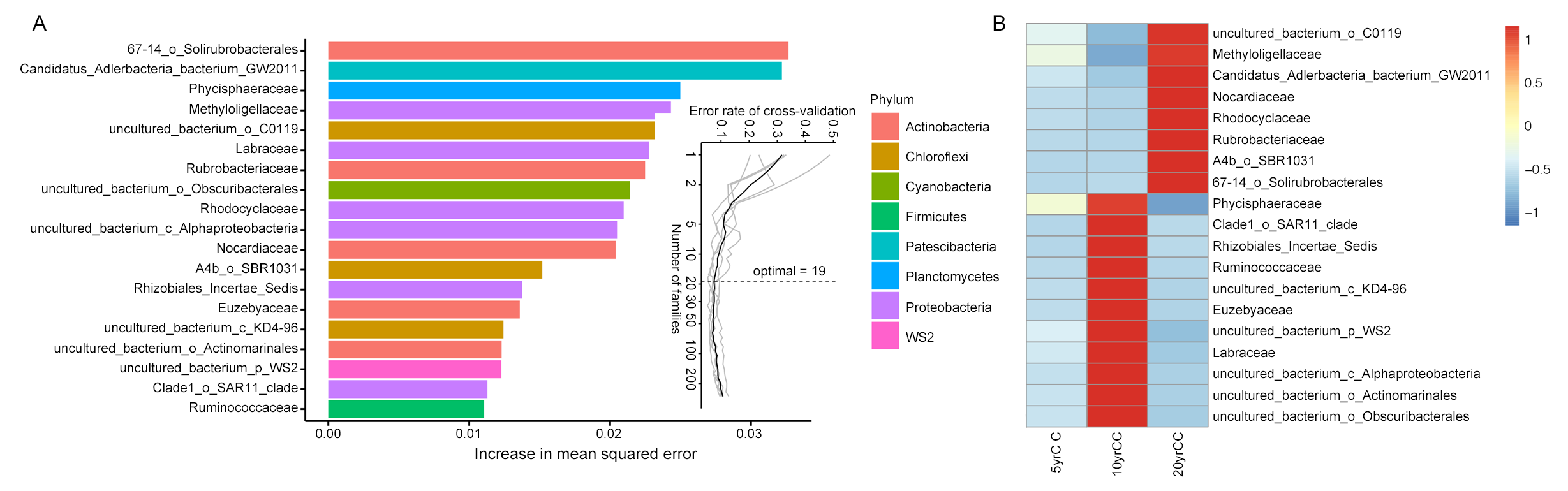

Supplement: Supplementary Figure 8 — Random-forest model to detect bacterial taxa representing the biomarkers across corresponding continuous monocropping time spans of flue-cured tobacco as detected by the rhizosphere bacterial community in the period of tobacco harvest. [file Image_8.TIF]
